# Supplementary material for: Replication stress triggers microsatellite destabilization and hypermutation leading to clonal expansion in vitro
Source: Nat Commun. 2019 Sep 2;10:3925. doi: 10.1038/s41467-019-11760-2 (PMC6718401; doi:10.1038/s41467-019-11760-2)
Supplement: Supplementary file 1 — Supplementary Information [file 41467_2019_11760_MOESM1_ESM.pdf]

Supplementary information for

Replication stress triggers microsatellite destabilization and hypermutation, leading to clonal expansion *in vitro*

Matsuno et al.

This PDF file includes:

Supplementary Fig. 1–7

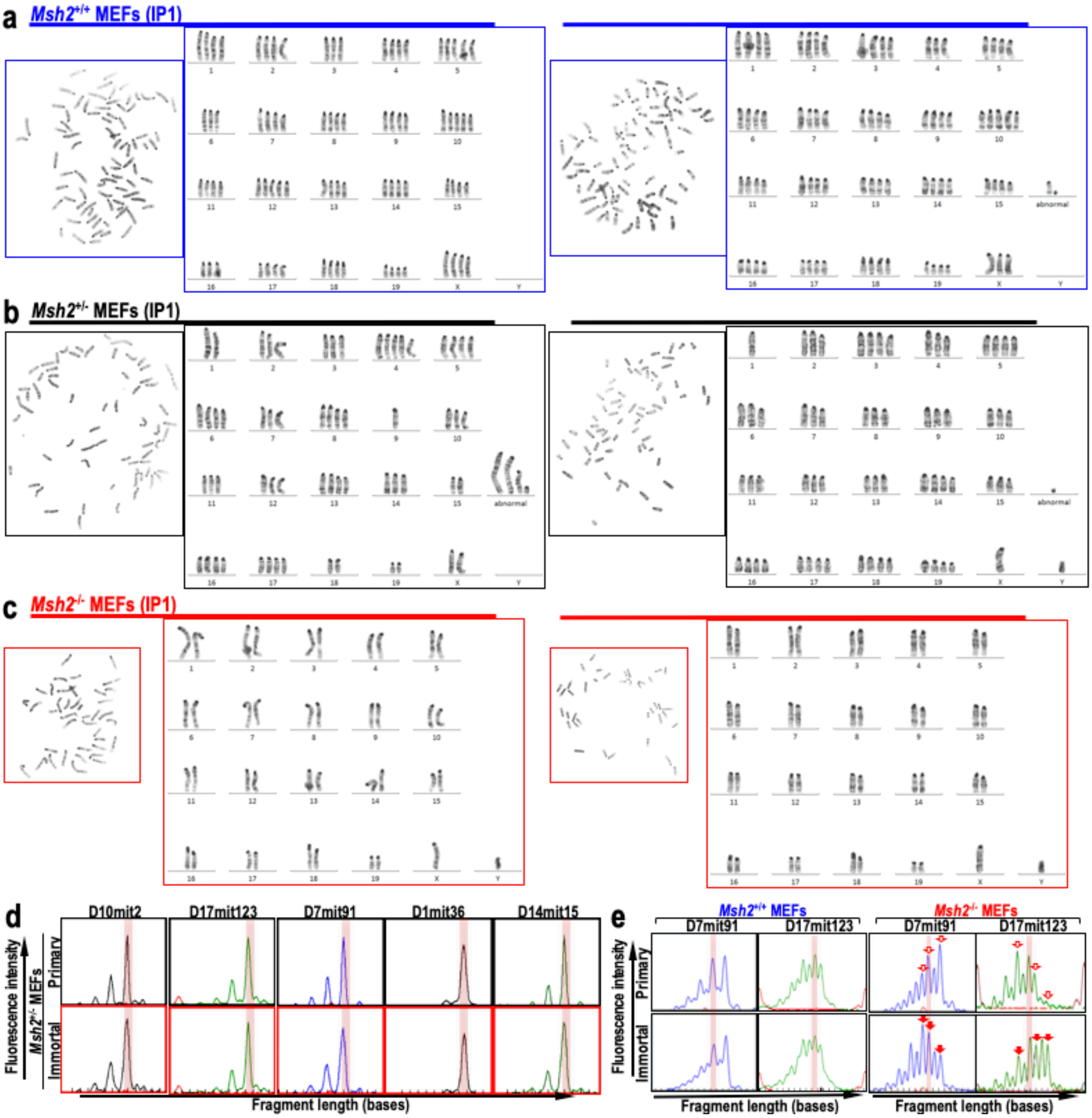

Supplementary Fig. 1. MMR-deficient MEFs immortalize without CIN. **a–c,** Representative chromosome images and karyotyping of immortalized MMR-deficient and -proficient MEFs. Representative images of chromosome spreads are shown for immortalized M-phase *Msh2*$^{+/+}$ (**a**), *Msh2*$^{+/-}$ (**b**), and *Msh2*$^{-/-}$ (**c**) MEFs, together with karyotyping. **d**, **e,** The MSI status of *Msh2*$^{+/-}$ MEFs was determined by comparing the fragment lengths at five MSI loci (D10mit2, D17mit123, D7mit91, D1mit36, and D14mit15) (**d**). The MSI statuses of *Msh2*$^{+/+}$ and *Msh2*$^{-/-}$ MEFs were determined by comparing the fragment lengths at D7mit91 and D17mit123 (**e**). Red arrows indicate the shifted fragment peaks at the MSI locus.

a
b
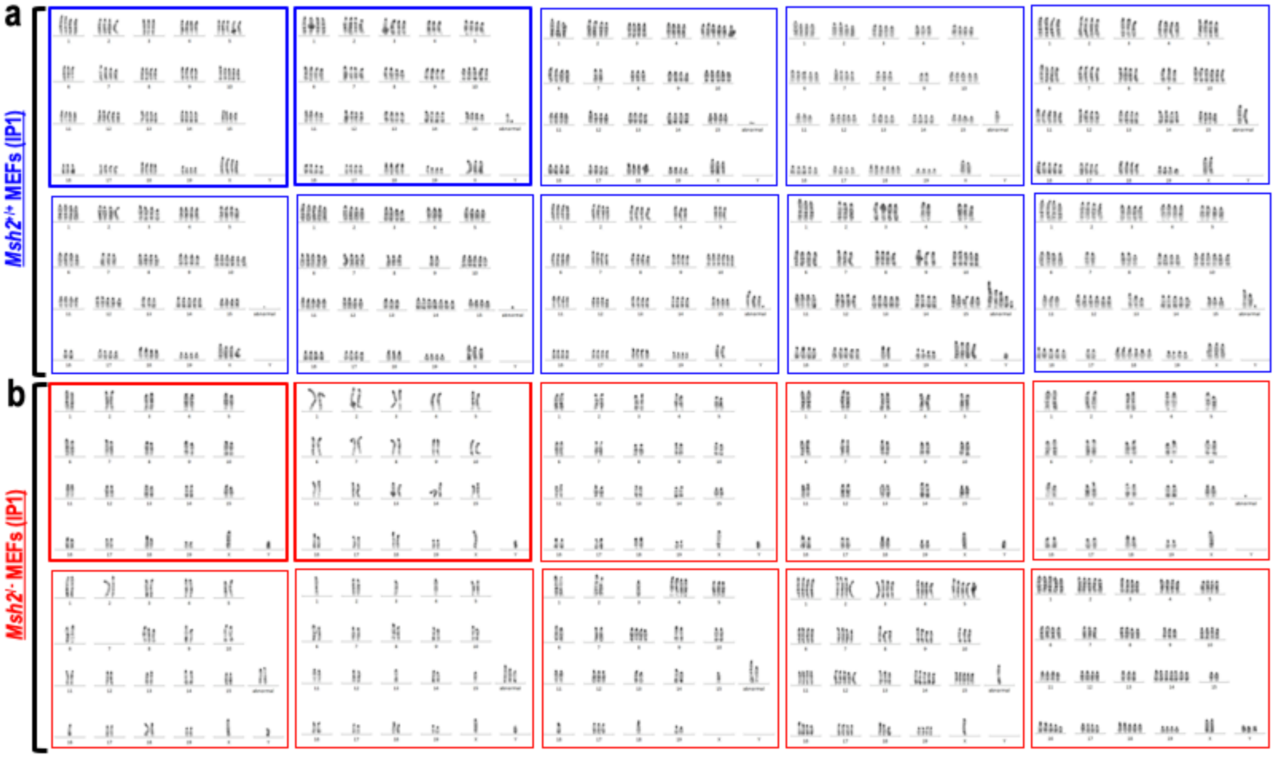

c

Chromosomal abnormalities	Numbers abnormalities observed in each nuclear in *Msh2*+/+ MEFs										Numbers abnormalities observed in each nuclear in *Msh2*-/- MEFs									
Tetraploidy	1	1	1	1	1	1	1	1	1	1	0	0	0	0	0	0	0	0	1	1
Numerical abnormalities other than tetraploidy	6	3	8	13	8	8	14	6	13	17	0	0	0	0	0	4	5	13	9	13
Other abnormal chromosomes	0	2	2	1	2	1	1	4	5	3	0	0	0	0	1	2	3	2	1	0
Others	0	0	0	0	0	0	0	0	1	0	0	0	0	0	0	0	0	0	0	0
Total numbers of abnormalities	7	6	11	15	11	10	16	11	20	21	0	0	0	0	1	6	8	15	11	14

d
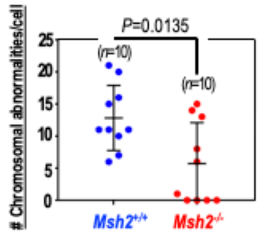

e
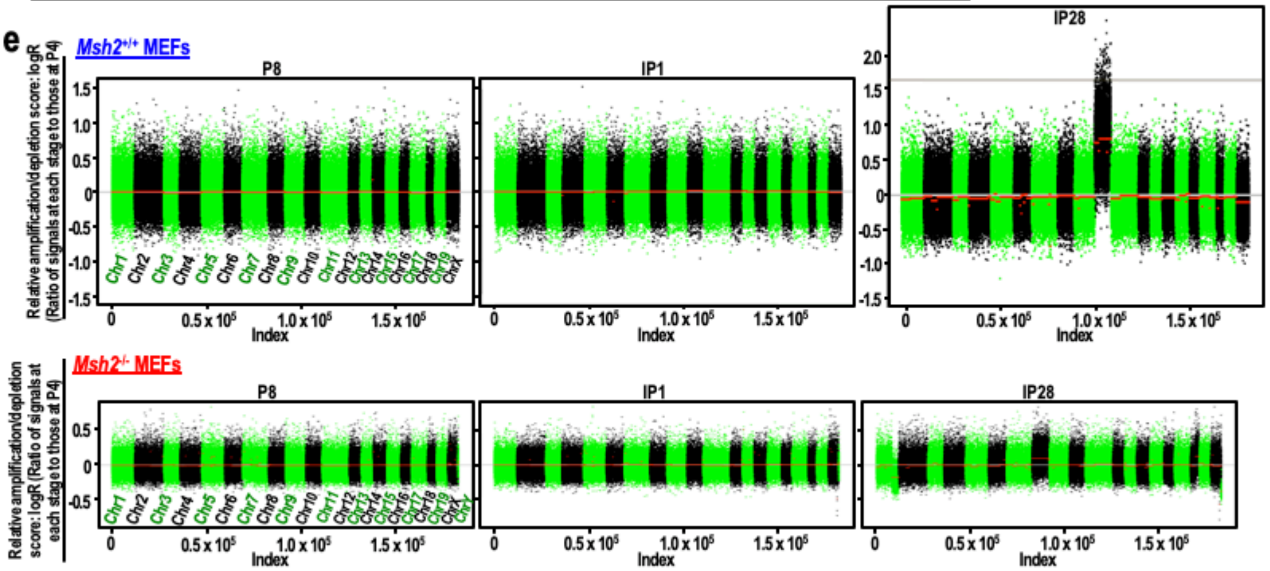

Supplementary Fig. 2. MMR-deficient MEFs exhibit minor chromosomal abnormalities. **a, b,** Karyotyping of chromosomes of *Msh2*$^{+/+}$ (**a**) and *Msh2*$^{-/-}$ MEFs (**b**). **c, d,** Chromosomal abnormalities were counted in each abnormality type (**c**) and plotted as total abnormality numbers for each nucleus (**d**,

n numbers are indicated in graph). Bars show means ± s.d. Two-tailed Welch's *t*-test was used for statistical analysis. **e,** Apparent copy number alterations at each exon locus were analyzed for exome data of $Msh2^{+/+}$ and $Msh2^{-/-}$ MEFs at each stage. Each exon locus was ordered on the x-axis from Chromosome 1 (left) to X/Y (right). Distributions of relative read numbers are plotted as green or black dots. Red dots represent the averages of each exon locus.

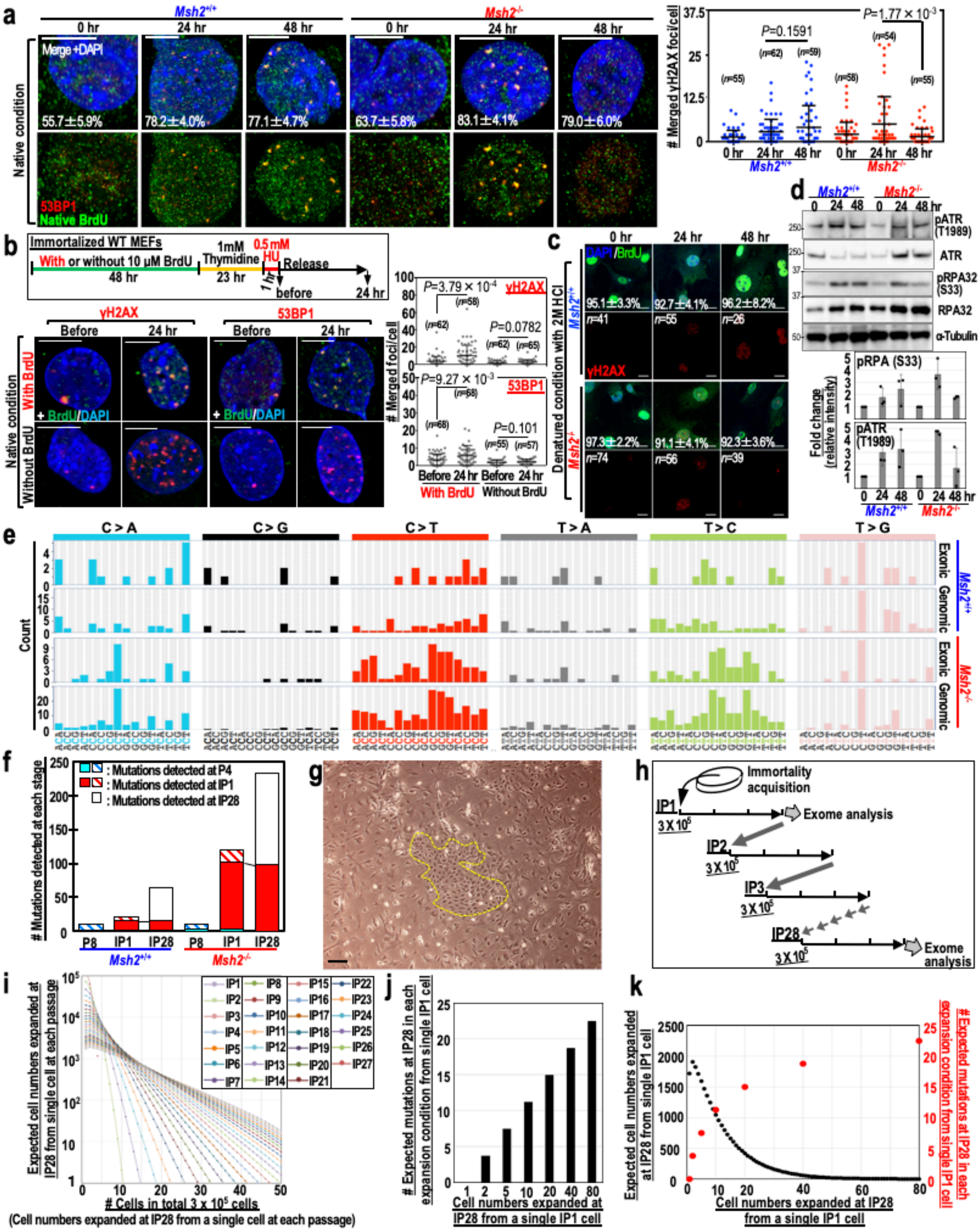

Supplementary Fig. 3. Hypermutation is induced before MMR-deficient MEFs immortalize. **a–c,** *Msh2*$^{+/+}$ and *Msh2*$^{-/-}$ MEFs were treated as shown in Fig. 2c. Foci of 53BP1 and BrdU under native conditions were detected by immunofluorescence (**a**, *n* numbers are indicated in graph). Percentages of

53BP1 foci colocalized with native BrdU foci (means ± s.e.) are indicated in each image. As control experiments of native BrdU staining, we performed experiments as in workflow and co-immuno-stained with anti-γH2AX, -53BP1 and -BrdU antibodies (**b**, *n* numbers are indicated in graph). To determine the BrdU incorporation status, BrdU signals under denatured conditions (incubation with 2M HCl) were detected by immunofluorescence along with γH2AX foci (**c**). Percentages of BrdU-positive cells visualized under this condition (means ± s.e.) are indicated in each image (**c**). Bars show means ± s.d. Scale bars, 10 μm. Two-tailed Welch's *t*-test was used for statistical analysis. **d,** Phosphorylation statuses of ATR and RPA in *Msh2*$^{+/+}$ and *Msh2*$^{-/-}$ MEFs were treated as shown in Fig. 2c. Signal intensities were normalized against α-tubulin ($n = 3$). Bars show means ± s.d. **e,** The spectra of mutations in immortalized *Msh2*$^{+/+}$ and *Msh2*$^{-/-}$ MEFs (IP28) were analyzed in the context of the flanking 1 bp sequence on each side. **f,** Mutations induced at different cellular stages, as identified by exome sequencing, are individually indicated. Light blue bars indicate mutations detected at P8 and carried over to IP1. Red bars indicate mutations detected at IP1 and carried over to IP28. **g,** Representative image of immortalized *Msh2*$^{-/-}$ MEFs. A colony of immortalized MEFs (indicated by the dashed yellow line) is surrounded by senescent cells with a flattened and enlarged morphology. Scale bar, 100 μm. **h,** Schematic illustration of the experiment. **i,** The numbers of MEFs expected to have expanded by IP28 from a single cell at the indicated stages. In this analysis, mutations were detectable when more than 3×10^4 cells accumulated. **j, k,** The numbers of possible mutations at IP28 were estimated under several expansion conditions. The expected number of mutations at IP28 depends on the expansion conditions during IP1–IP28 (**j**). Estimations are based on the assumption that all resulting cells expanded equally. The expected numbers of mutations (red dots) and the expected number of cells expanded at IP28 from a single MEF at IP1 (black dots) are plotted (**k**).

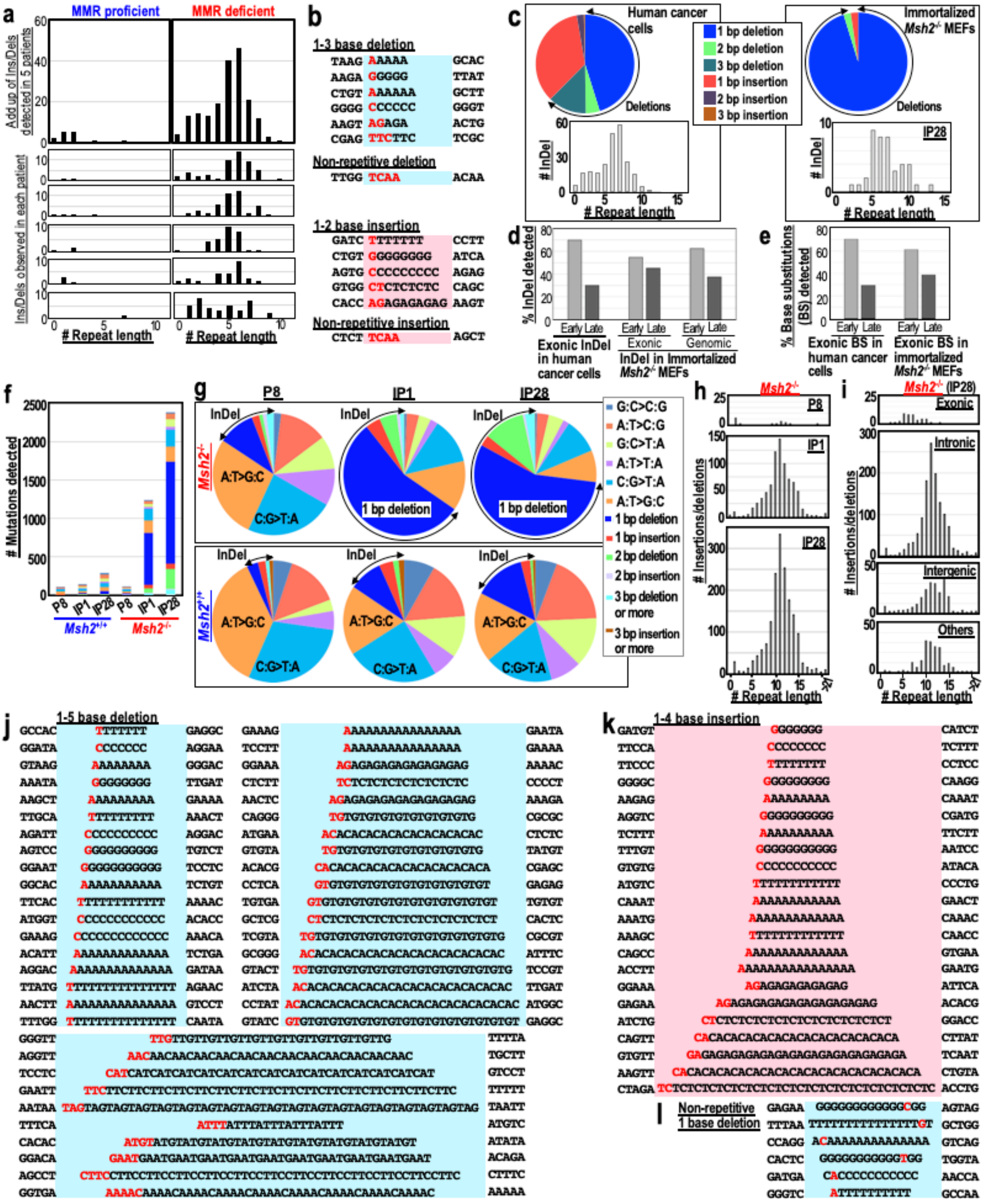

Supplementary Fig. 4. Sequences around inserted/deleted bases in human colorectal cancers and immortalized *Msh2*$^{-/-}$ MEFs. **a, b,** Insertion/deletion mutations (InDels) were analyzed in MMR-proficient and -deficient colorectal cancers (five patients in each group) randomly picked from TCGA

(bottoms) and their add-ups (Top). Repeat numbers at the detected InDels were analyzed (**a**). Repetitive sequences around inserted/deleted bases in MMR-deficient colorectal cancer cells (**b**). A value of 0 indicates no repeats. **c,** The InDel spectra of MMR-deficient colorectal cancer cells and MMR-deficient MEFs were compared. Arrows indicate deletion mutations. **d, e,** Localizations of the detected InDels (**d**) and base substitutions (**e**) were analyzed by the genomic regions that are replicated at the indicated S-phase stages. **f, g,** Mutations detected in whole sequence data were analyzed at each cellular stage. The numbers (**f**) and spectra (**g**) of mutations in MEFs at each cellular stage are shown according to the indicated types. The arrowed regions indicate insertion/deletion mutations (InDel). **h**, **i,** The numbers of insertions and deletions were analyzed in simple repeats at each cellular stage (**h**) and in various genomic locations (**i**). The x-axis indicates the number of repetitive subunits surrounding an insertion or deletion. A value of 0 indicates no repeats. **j–l,** Representative sequences around 1–5 base deletions (**j**) and 1–4 base insertions (**k**), together with 1 base deletion in non-repetitive bases (**l**), are shown. Deleted/inserted bases are indicated by red letters.

a

MSI status	MSI-L	MSI-H	MSS	NA
Total number	44	64	187	0
CDKN2A deletion	5	2	22	0
CDKN2A mutation	11	29	37	0
TP53 deletion	1	2	1	0
TP53 mutation	26	25	87	0

b

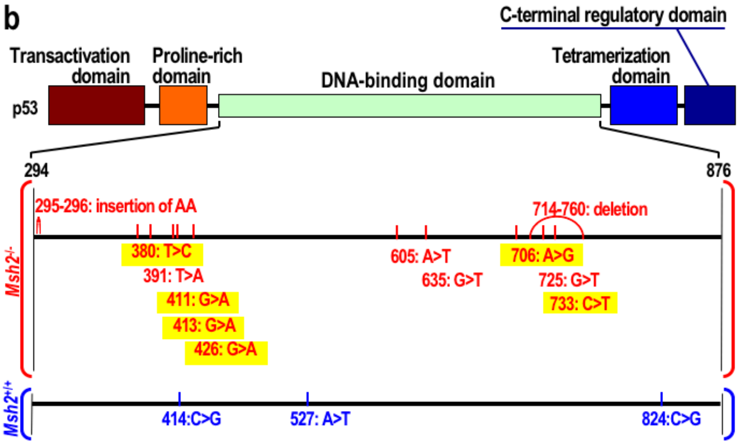

Supplementary Fig. 5. Deletion and point mutations arise in the ARF/p53 module. **a,** Numbers of each type of mutation in gastric cancers from the TCGA are indicated according to the MSI/MSS (microsatellite stability) statuses. **b,** p53-DBD cDNA was cloned from independently immortalized *Msh2*$^{-/-}$ and *Msh2*$^{+/+}$ MEFs. Mutations detected in the p53 DBD are shown. Mutations inducible with GT-mismatches are marked in yellow (C>T and A>G). MSI-H, MSI-high; MSI-L, MSI-low.

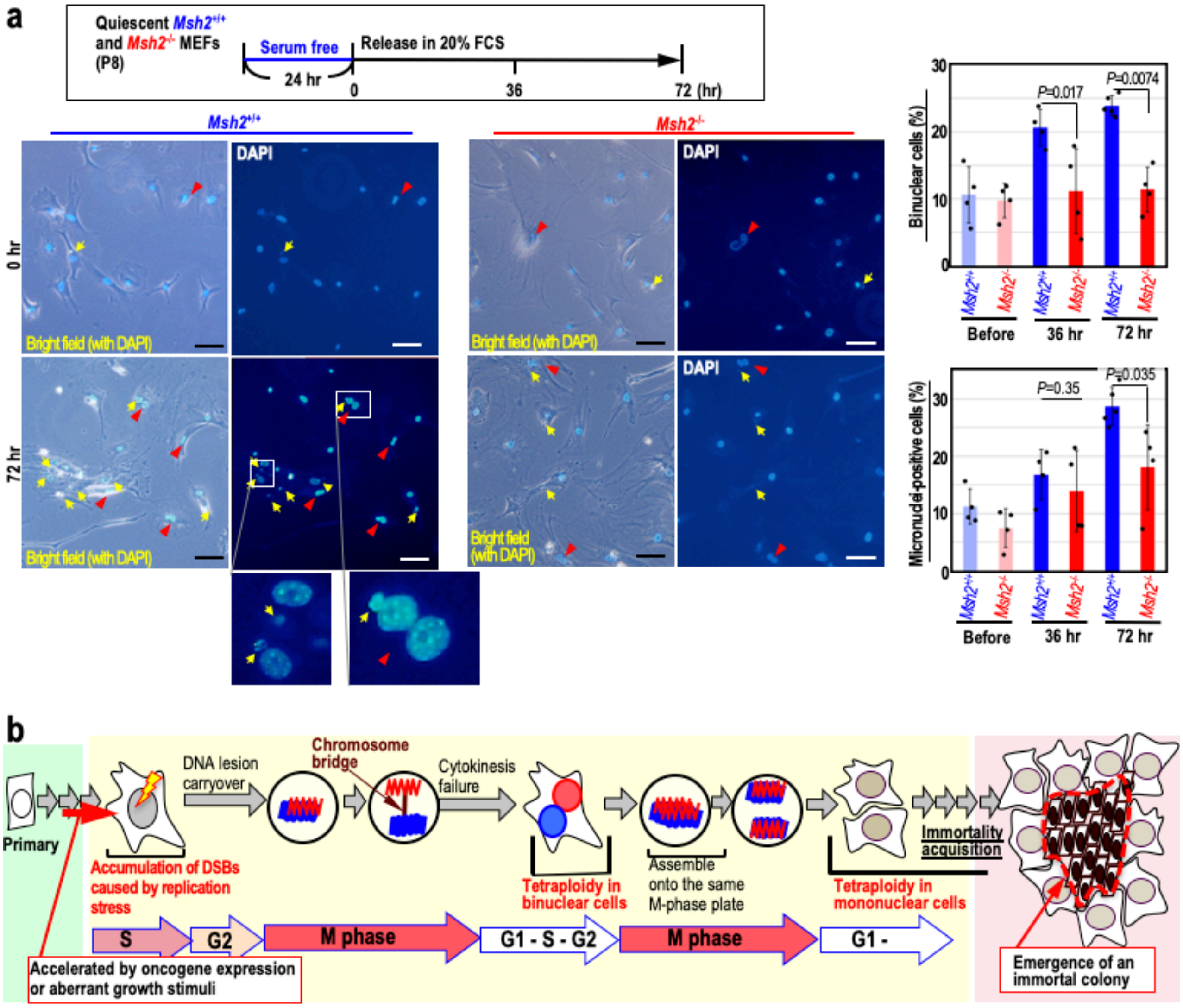

Supplementary Fig. 6. Replication stress-triggered tetraploidization initially induces the formation of binuclear cells following cytokinesis failure. **a,** $Msh2^{-/-}$ and $Msh2^{+/+}$ MEFs were cultivated under the workflow shown in the upper box. Binuclear cells (red arrowheads) and micronuclei formation (yellow arrowheads) were observed. Quantification of these cells at the indicated time points is shown in the right panels (n = 4 independent experiments). Bars show means ± s.d. Scale bars, 100 µm. Two-sample test for equality of proportions with continuity correction was used for statistical analysis. **b,** Model of the tetraploidization process before $Msh2^{+/+}$ MEFs acquire immortality. Aberrant growth stimulation leads to the accumulation of replication stress-associated DSBs. DSBs carried over into M-phase mediate chromosomal bridge formation, resulting in tetraploidization in association with cytokinesis failure. This leads to tetraploidy in binuclear cells until the following M-phase, during which chromosomes assemble on the same M-phase plate, causing tetraploidy in mononuclear cells in the subsequent G1 phase.

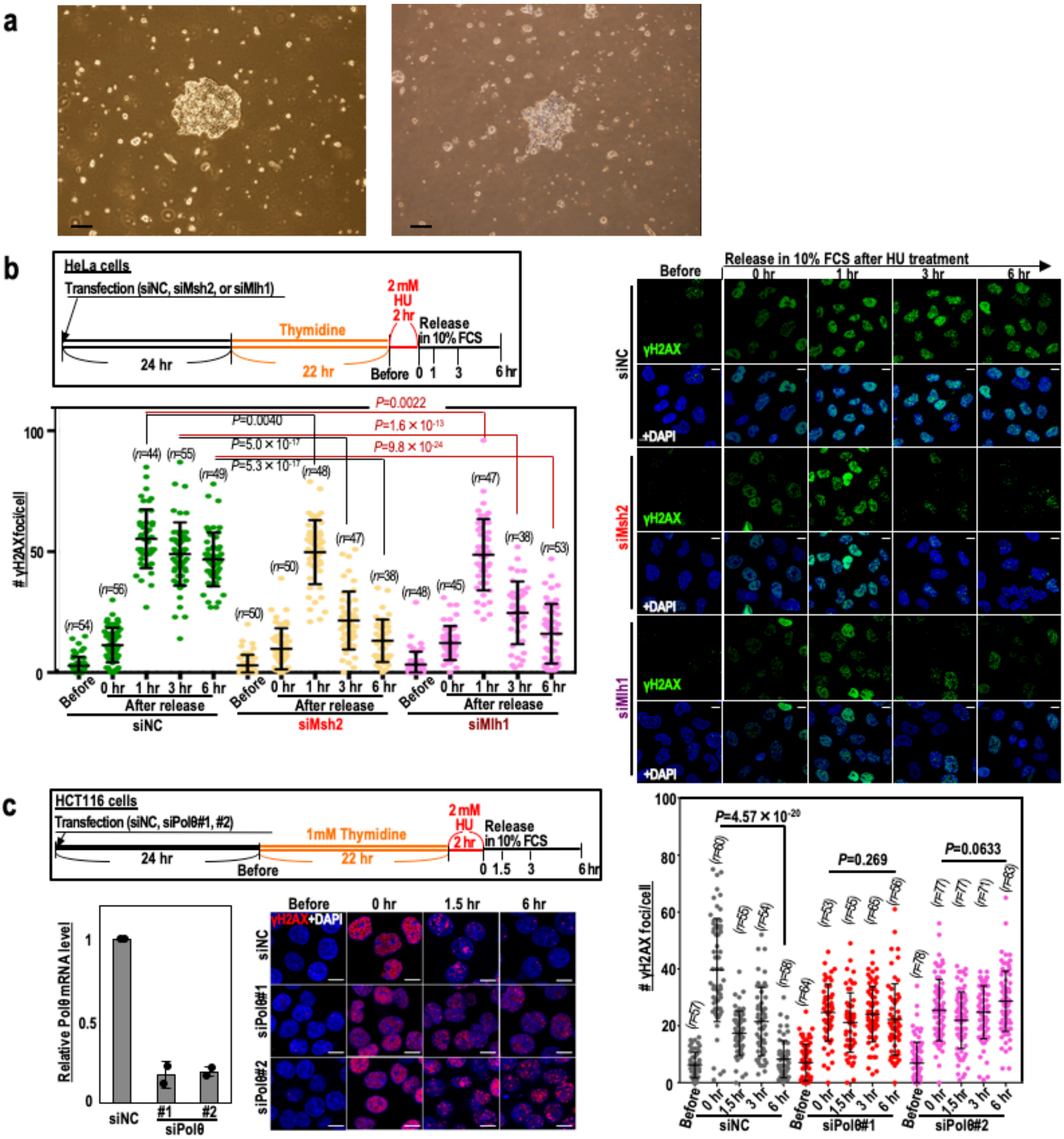

Supplementary Fig. 7. Polθ and PARP mediate repair of replication stress-associated DSBs in an MMR-deficient background. **a,** Representative images of cancer cells that recovered growth activity after CPT treatment: HCT116 (left); HeLa (right). CPT-resistant cells appeared as colonies surrounded by cells undergoing to apoptosis or senescence. Scale bar, 100 µm. **b,** Repair of replication stress-associated DSBs is compromised in MMR-deficient cells after cells were treated as shown in the workflow. MSH2- and MLH1-KD HeLa cells in S phase were pulse-treated with HU as shown in the upper box. γH2AX foci in cells released into HU-free medium were assessed by immunofluorescence microscopy. Quantification of the number of foci per nucleus is also shown (*n* numbers are indicated in graph). Bars show means ± s.d. Scale bars, 10 µm. Two-tailed Welch's *t*-test was used for statistical

analysis. Replication stress-induced DSBs were efficiently repaired by Polθ in HCT116 cells. **c,** Control and Polθ-knockdown HCT116 cells were treated with HU as shown in the upper box. Knockdown of Polθ was confirmed by comparison with negative control cells (left panel, $n = 2$). Decay rates of γH2AX foci were monitored by immunofluorescence. Quantification of the number of foci per nucleus is also shown (n numbers are indicated in graph). Bars show means ± s.d. Scale bars, 10 μm. Two-tailed Welch's t-test was used for statistical analysis.
