# Supplementary material for: Replication stress triggers microsatellite destabilization and hypermutation leading to clonal expansion in vitro
Source: Nat Commun. 2019 Sep 2;10:3925. doi: 10.1038/s41467-019-11760-2 (PMC6718401; doi:10.1038/s41467-019-11760-2)
Supplement: Supplementary file 3 — Reporting Summary [file 41467_2019_11760_MOESM3_ESM.pdf]

nature research

Corresponding author(s): Ken-ichi Yoshioka

Last updated by author(s): Jul 26, 2019

Reporting Summary

Nature Research wishes to improve the reproducibility of the work that we publish. This form provides structure for consistency and transparency in reporting. For further information on Nature Research policies, see Authors & Referees and the Editorial Policy Checklist.

Statistics

For all statistical analyses, confirm that the following items are present in the figure legend, table legend, main text, or Methods section.

n/a	Confirmed	
☐	☒	The exact sample size (*n*) for each experimental group/condition, given as a discrete number and unit of measurement
☐	☒	A statement on whether measurements were taken from distinct samples or whether the same sample was measured repeatedly
☐	☒	The statistical test(s) used AND whether they are one- or two-sided<br>*Only common tests should be described solely by name; describe more complex techniques in the Methods section.*
☒	☐	A description of all covariates tested
☐	☒	A description of any assumptions or corrections, such as tests of normality and adjustment for multiple comparisons
☐	☒	A full description of the statistical parameters including central tendency (e.g. means) or other basic estimates (e.g. regression coefficient) AND variation (e.g. standard deviation) or associated estimates of uncertainty (e.g. confidence intervals)
☐	☒	For null hypothesis testing, the test statistic (e.g. *F*, *t*, *r*) with confidence intervals, effect sizes, degrees of freedom and *P* value noted<br>*Give P values as exact values whenever suitable.*
☒	☐	For Bayesian analysis, information on the choice of priors and Markov chain Monte Carlo settings
☒	☐	For hierarchical and complex designs, identification of the appropriate level for tests and full reporting of outcomes
☒	☐	Estimates of effect sizes (e.g. Cohen's *d*, Pearson's *r*), indicating how they were calculated

Our web collection on statistics for biologists contains articles on many of the points above.

Software and code

Policy information about availability of computer code

Data collection	EC800 software(Sony), FV10i software(Olympus) and LAS4000 software(GE healthcare) were used.
Data analysis	Excel, Prism7, ImageJ, CEQ8000, EC800 and UCSC Genome Browser were used.

For manuscripts utilizing custom algorithms or software that are central to the research but not yet described in published literature, software must be made available to editors/reviewers. We strongly encourage code deposition in a community repository (e.g. GitHub). See the Nature Research guidelines for submitting code & software for further information.

Data

Policy information about availability of data

All manuscripts must include a data availability statement. This statement should provide the following information, where applicable:

- Accession codes, unique identifiers, or web links for publicly available datasets
- A list of figures that have associated raw data
- A description of any restrictions on data availability

Exome analysis data are available, described in Methods.

Field-specific reporting

Please select the one below that is the best fit for your research. If you are not sure, read the appropriate sections before making your selection.

☒ Life sciences ☐ Behavioural & social sciences ☐ Ecological, evolutionary & environmental sciences

For a reference copy of the document with all sections, see nature.com/documents/nr-reporting-summary-flat.pdf

Life sciences study design

All studies must disclose on these points even when the disclosure is negative.

Sample size	Experiments were conducted on 3 or more independent biological samples.
Data exclusions	No data were excluded.
Replication	All experimental findings were reliably reproduced as indicated in the figure legends.
Randomization	Randomization was not performed.
Blinding	Blinding was not performed.

Reporting for specific materials, systems and methods

We require information from authors about some types of materials, experimental systems and methods used in many studies. Here, indicate whether each material, system or method listed is relevant to your study. If you are not sure if a list item applies to your research, read the appropriate section before selecting a response.

Materials & experimental systems

n/a	Involved in the study	
☐	☒	Antibodies
☐	☒	Eukaryotic cell lines
☒	☐	Palaeontology
☒	☐	Animals and other organisms
☒	☐	Human research participants
☒	☐	Clinical data

Methods

n/a	Involved in the study	
☒	☐	ChIP-seq
☐	☒	Flow cytometry
☒	☐	MRI-based neuroimaging

Antibodies

Antibodies used	Validated antibodies against the following proteins were obtained from the indicated suppliers: 53BP1 (PC712, Merck), α-tubulin (T6074, Sigma), β-actin (AC-74, Sigma), H3(MABI0301, MBL), γH2AX (9718, Cell Signaling), PCNA (ab29, Abcam), ATR(sc-515173, Santa Cruz), phospho-ATR (Thr1989) (GTX128145, GeneTex), RPA32 (2208, Cell Signaling), phospho-RPA32 (Ser33) (E-AB-21080, Elabscience), Polδ (ab129498, Abcam), Polη (A301-231A, Bethyl; Ohkumo et al., 2006), Polι (Ohkumo et al., 2006), Polκ (generated in rabbit and purified with a Protein G column), Rad51 (8875, Cell Signaling) and BrdU (66241-1-lg, proteintech).
Validation	All antibodies were validated by the producers.

Eukaryotic cell lines

Policy information about cell lines

Cell line source(s)	HeLa and HCT116 were obtained from ATCC.
Authentication	Cell lines were authenticated by JCRB Cell Bank.
Mycoplasma contamination	Cells were mycoplasma negative.
Commonly misidentified lines (See ICLAC register)	No commonly misidentified cell line was used.

Flow Cytometry

Plots

Confirm that:

☒ The axis labels state the marker and fluorochrome used (e.g. CD4-FITC).

☒ The axis scales are clearly visible. Include numbers along axes only for bottom left plot of group (a 'group' is an analysis of identical markers).

☒ All plots are contour plots with outliers or pseudocolor plots.

☒ A numerical value for number of cells or percentage (with statistics) is provided.

Methodology

Sample preparation	Collected cells were incubated with PBS containing 0.1% Triton X-100 and 100 ug/mL RNase A for 30 minutes at RT and centrifuged. Centrifuged cells were re-incubated with PBS containing 10 ug/mL propidium iodide, 0.1% Triton X-100 and 100 ug/mL RNase A for 30 minutes at RT.
Instrument	Data were acquired by using EC800 (SONY).
Software	Data were analyzed by using EC800 software (SONY).
Cell population abundance	N/A
Gating strategy	All cells were analyzed.

☒ Tick this box to confirm that a figure exemplifying the gating strategy is provided in the Supplementary Information.
